# Natural Colorants From Amazonian Plants: Bioactive Potential and Sustainable Extraction Techniques—A Review

**DOI:** 10.1002/fsn3.71772

**Published:** 2026-05-14

**Authors:** Zandleme Birino de Oliveira, Kamila Brielle Pantoja Vasconcelos, Amanda de Lima Silva Mumberger, Antônio Quaresma da Silva Junior, Leomara Andrade da Silva, Ricardo Bezerra de Oliveira, Rosa Helena Veras Mourão, Sandra Layse Ferreira Sarrazin

**Affiliations:** ^1^ Graduate Program in Biodiversity and Biotechnology – BIONORTE Network Santarém Brazil; ^2^ Graduate Program in Health Sciences (PPGSA) Graduate Program in Biodiversity and Biotechnology – BIONORTE Network Santarém Brazil

**Keywords:** Amazon, green solvents, natural dyes, sustainable extraction

## Abstract

This review synthesized and compared extraction methodologies for anthocyanins, carotenoids, betalains, and chlorophylls from Amazonian species, highlighting extraction yield, compound stability, bioactivity preservation, and industrial feasibility of conventional and green approaches. A systematized search was conducted in Google Scholar, ScienceDirect, PubMed, SciELO, ACS Publications, and Scopus, covering publications from 2010 to 2025. Eligible studies included original articles, systematic reviews, and laboratory assays reporting extraction techniques with analytical identification confirmed by high‐performance liquid chromatography (HPLC) or spectrophotometry and quantitative yield data. In total, 36 records of Amazonian species were analyzed: anthocyanins (*n* = 12), carotenoids (*n* = 9), betalains (*n* = 6), and chlorophylls (*n* = 10), in addition to 18 comparative studies involving other plant matrices. açaí ( *Euterpe oleracea* ), bacaba (*Oenocarpus bacaba*), and camu‐camu (*Myrciaria dubia*) were the main sources of anthocyanins, whereas buriti ( *Mauritia flexuosa* ) and tucumã (*Astrocaryum vulgare*) accounted for most carotenoid reports. Betalains were primarily described in pitaya and facheiro, and chlorophylls in tree leaves. Deep eutectic solvents (DES), natural deep eutectic solvents (NADES), and supercritical carbon dioxide (CO_2_), combined with ultrasound‐assisted extraction (UAE), microwave‐assisted extraction (MAE), and pulsed electric fields (PEF), showed superior performance compared with conventional methods. DES containing choline chloride achieved 41.3 mg/100 g of carotenoids versus 11.5 mg/100 g with ethanol, being up to 3.5 times more efficient. The PEF–ethanol combination recovered more than 80% of total carotenoids. Green solvents associated with emerging technologies represent technically viable and environmentally superior routes, while native betalains and chlorophylls remain underexplored, representing a priority research gap.

## Graphical abstract

This review explores Amazonian plants as sources of natural colorants, highlighting major groups such as anthocyanins, carotenoids, betalains, and chlorophylls. It emphasizes sustainable extraction techniques, including green solvents and emerging technologies, and discusses their bioactive properties and potential applications in the food industry.

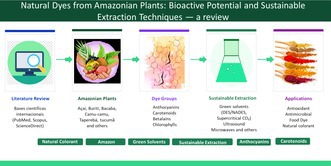

## 1 Introduction

In recent decades, the food chain has undergone a process of substantial reformulation, driven mainly by the rise in collective awareness around the promotion of health, well‐being, and sustainability (Cazón 2024). Among the main contemporary challenges, the need to reduce dependence on synthetic additives widely used by the food industry stands out with the aim of giving color, flavor, texture, and a longer shelf life to products (Cano‐Lamadrid and Artés‐Hernández 2022).

At the same time, there is a transformation in consumption habits, characterized by a growing preference for natural, minimally processed foods with more transparent labels. This trend, often called clean label, reflects the appreciation of ingredients of recognized origin, free of controversial substances and with added functional appeal (Andrade et al. 2024). In this new paradigm, the nutritional profile, traceability, and bioactivity of compounds begin to play a leading role in purchasing decisions (Eck and Byrd‐Bredbenner 2020).

Despite the potential of Amazonian flora to produce natural compounds for use in the food industry, the development and application of sustainable extraction technologies in this region remain incipient, which limits the incorporation of these additives into food systems guided by principles of naturalness and sustainability. Regarding sustainability, green technologies add value to products from companies committed to environmentally responsible practices and, consequently, strengthen the Amazonian bioeconomy; however, the limited availability of investment and the insufficient public policies aimed at fostering regional entrepreneurship still constitute significant barriers to the consolidation of this sector (Freitas et al. 2024).

Specifically regarding food coloration, artificial colorants have been widely questioned, particularly due to studies associating them with adverse reactions such as gastrointestinal disorders, childhood hyperactivity, allergic responses, and even carcinogenic effects (Oliveira et al. 2024). Nevertheless, visual appeal remains one of the main criteria for consumer acceptance, as consumers tend to associate color intensity with product quality and freshness (Sharmila 2019). Given this premise, there is an urgent need to identify safe, efficient, and sustainable alternatives for food coloring (Tzanova et al. 2024).

In this scenario, natural colorants of plant origin have established themselves as promising technological alternatives. In addition to their ability to impart attractive coloration, these compounds are often associated with high‐value‐added functional properties, such as antioxidant activity (Barros et al. 2017), biological actions against cancer (Rodriguez‐Concepcion et al. 2018), antimicrobial effects (de Jesus et al. 2020), and chemoprotective activity (Loypimai and Moongngarm 2017). At the same time, increased awareness of the risks associated with the use of artificial colors has stimulated the development of more environmentally friendly extraction methodologies, such as so‐called green technologies, which aim to optimize the use of natural solvents and reduce environmental impacts, without compromising process efficiency (Tzanova et al. 2024).

From this perspective, the strategic potential of the Amazon region stands out, recognized worldwide as one of the largest reservoirs of biodiversity on the planet. This vast and rich flora is home to a myriad of plant species, both fruit‐bearing and non‐fruit‐bearing, many of which remain little explored in terms of their phytochemical composition and potential for industrial application (Chisté et al. 2021). In this context, Brazil assumes a leading role in prospecting for bioactives of technological and nutritional interest.

Amazonian species such as açaí ( *Euterpe oleracea* ), bacaba (*Oenocarpus bacaba*), buriti ( *Mauritia flexuosa* ), and camu‐camu (*Myrciaria dubia*) have been reported as promising sources of natural colorants with technological application potential. In açaí and bacaba, anthocyanins and other phenolic compounds predominate, mainly concentrated in the pulp and peel (Domingues et al. 2012; Abadio Finco et al. 2012, respectively). Buriti is characterized by carotenoids with high provitamin A activity (Santos et al. 2015), whereas camu‐camu is rich in anthocyanins associated with high vitamin C content (Neves et al. 2015).

This diversity of Amazonian fruits highlights that pigment distribution varies widely according to compound class and plant matrix, directly implying different extraction strategies and biomass utilization approaches (Miranda et al. 2021; Villa‐Hernández et al. 2017).

The chemical nature of colorants directly determines the choice of extraction system. Anthocyanins and betalains, which are water‐soluble compounds, are efficiently recovered using polar solvents such as ethanol, methanol, and acidified water. In contrast, carotenoids, which are lipophilic, require nonpolar solvents or strategies such as supercritical fluid extraction (SFE) (Mattioli et al. 2020; Morón‐Ortiz et al. 2024).

Chlorophylls exhibit intermediate behavior and are commonly extracted with acetone or ethanol. Although conventional methods (maceration, solid–liquid extraction, Soxhlet) are still widely employed, their efficiency is often limited by high solvent consumption, thermal degradation of compounds, and low selectivity.

In this context, emerging technologies including ultrasound‐assisted extraction (UAE), microwave‐assisted extraction (MAE), enzyme‐assisted extraction (EAE), pulsed electric fields (PEF), and natural deep eutectic solvents (NADES/DES) have demonstrated superior performance in terms of extraction yield, bioactivity preservation, and reduced environmental impact (Tzanova et al. 2024; Miranda et al. 2021).

This review is justified by the need to fill specific gaps related to the limited integration between the bioactive potential of Amazonian colorants and their technological application in sustainable food systems. Although the Amazon region harbors extensive plant diversity capable of producing natural colorants, systematic analyses correlating bioactivity, stability, and extraction feasibility from a green technology perspective remain scarce.

In this context, the present review organizes the available evidence according to the following analytical structure: (i) predominant colorants classes in Amazonian biodiversity (anthocyanins, carotenoids, betalains, and chlorophylls); (ii) associated plant matrices (fruits, leaves, branches, stems, and flowers); (iii) the most suitable conventional and green extraction routes (DES/NADES and supercritical CO_2_); and (iv) potential applications in the food industry, including a critical comparison between conventional and green solvents, as well as among emerging techniques, highlighting efficiency, sustainability, and industrial feasibility.

## 2 Methodology

The present integrative review was developed based on the collection and analysis of scientific publications available in recognized national and international databases, including Google Scholar, ScienceDirect, PubMed, SciELO, ACS Publications, and Scopus. The systematic search was conducted with the aim of gathering updated evidence on the main extraction methods of natural colorants, with an emphasis on plant matrices from the Amazon region. In this work, plants recognized as producers of natural dyes were prioritized, also considering the bioactive potential of these substances. This approach allowed the selection of species that, in addition to providing coloration, may also exhibit relevant biological properties, expanding their application prospects in food and functional contexts.

To retrieve publications, descriptors and keyword combinations were used, such as: *pigment extraction methods*, *natural colorants*, *Amazonian plant colorants*, *Amazonian fruit colorants*, *exotic fruits of the amazon*, *carotenoids*, *anthocyanins*, *betalains, chlorophylls*, and *green solvents*. The filters applied prioritized original articles, systematic reviews, experimental studies, and laboratory tests published in the last 15 years.

**FIGURE 1:**
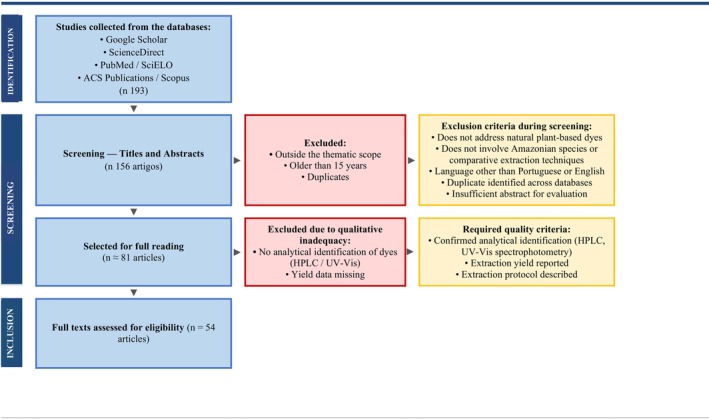
Flowchart of the methodology for the bibliographic survey on natural colorants and extraction solvents in Amazonian fruits and plants.

The selection of material followed criteria of scientific relevance, timeliness, methodological clarity, and pertinence to the central theme of the research. After an initial screening of titles and abstracts, the eligible articles were read in full for data extraction, particularly concerning the extraction techniques, the efficiency of the solvents used, the phytochemical composition of the investigated species, and the applicability of the colorants in the food industry (Figure 1).

## 3 Sources of Colorants in Amazonian Plants

Quantitative analysis of the collected publications shows that different groups of colorants have been reported in Amazonian flora species. The compiled tables highlight 11 records of anthocyanin sources, distributed primarily in economically important fruits, which also stand out as sources of this group of colorants (Barbosa et al. 2019; Neves et al. 2015; Dos Santos et al. 2015; de Oliveira 2019; Menezes et al. 2015). In the case of carotenoids, nine records were identified, with emphasis on oleaginous and carotenogenic fruits (Santos et al. 2015; Amorim et al. 2022; Chisté et al. 2021). Significant concentrations of species such as taperebá ( *Spondias mombin* ) (Costa and Mercadante 2018) and araçá‐boi (*Eugenia stipitata*) (Garzón et al. 2012) were also observed, confirming the relevance of this group in the region.

Betalains, although less frequent, were recorded in six species. Despite being less studied, these species highlight the potential for further exploration of this colorant group, particularly due to their vivid coloration and stability under specific conditions. (Mello et al. 2015; Rodriguez et al. 2016; Rodrigues et al. 2019; Dantas et al. 2016). Chlorophylls, in turn, were reported in ten records, mainly from the leaves of arboreal species (Lamarre et al. 2012; Marenco and de Freitas Sousa 2019; Mori et al. 2019), as well as fruits (Andrade Júnior et al. 2016; Barros et al. 2017). In summary, these numbers reinforce that studies are focused on anthocyanins and carotenoids, while betalains and chlorophylls remain relatively underexplored, revealing both the potential and gaps for future research.

### 3.1 Anthocyanins: Characteristics, Applications, and Natural Sources

Anthocyanins are molecules that belong to the flavonoid class, which also includes flavanols, flavanones, isoflavones, and flavones (Alseekh et al. 2020; Owona and Abia 2020). Anthocyanins constitute an important group of water‐soluble colorants whose coloration varies from pink, red, orange, and purple to blue, according to the pH of the surrounding medium (Tarone et al. 2020). These colorants not only impart attractive coloration to foods but also exhibit relevant bioactive properties, such as anti‐inflammatory activity (Owona and Abia 2020), antioxidant capacity (Abadio Finco et al. 2012), protection against diabetes (Guo and Ling 2015), and potential anticancer effects (Forbes‐Hernandez et al. 2016), which underscores their relevance for functional applications in the health and food sectors (Miranda et al. 2021).

One of the most relevant characteristics of these substances is their high pH sensitivity. According to Mattioli et al. (2020), in acidic environments (pH ≤ 3), anthocyanins tend to present a reddish coloration due to the predominance of the flavylic cationic form. As the pH approaches neutrality (6–7), conversion to anhydroquinoid structures occurs, conferring violet hues, while in weakly alkaline environments, the compounds may acquire a bluish coloration. However, at strongly basic pHs, the molecules become unstable, favoring their degradation.

Although this instability is sometimes regarded as a limitation for their use as natural colorants, Liu et al. (2018) highlight that this property can also be strategically exploited. The authors explain that the ability of anthocyanins to change color in response to pH variations can be used as a functional feature, especially in foods where acidity serves as a quality parameter, such as sauces, juices, yogurts, and processed meats.

In line with this perspective, Passeri et al. (2016) suggest that the presence of anthocyanins may act as a visual alert system: as pH changes occur, whether due to microbial degradation, oxidation, or undesirable fermentation, the color of the food also changes. This feature has been explored in the development of intelligent packaging, biodegradable indicator films, and formulations designed to provide greater transparency and safety to consumers (Zhao et al. 2022).

Regarding the natural distribution of these colorants, it is observed that anthocyanins are secondary metabolites widely produced by plants. A study evidenced their significant occurrence in plant species from the Amazon region, identifying notable anthocyanin contents in coramina (*Pedilanthus tithymaloides)* and mussambê ( *Tarenaya spinosa* ). Concentrations of 1.46 and 0.66 mg/g were reported for *P. tithymaloides* and 0.66 and 0.74 mg/g for *T. spinosa* , suggesting that variables such as the analyzed plant part, developmental stage, and environmental factors may directly influence the phytochemical composition of the samples (Vieira et al. 2022).

In the species *Pseudobombax marginatum* (embiratanha), significant amounts of anthocyanins were detected not only in the leaves but also in the branches, stem, roots, and flowers. This diversity in colorant localization demonstrates the potential of the species as a natural source of colorants. Moreover, since it is a plant used in traditional medicine, the interest in its bioactive constituents becomes even more relevant, broadening the prospects for application in different fields (Table 1) (Menezes et al. 2015).

**TABLE 1:** Amazonian plants identified as natural sources of anthocyanins.

Common name	Scientific name	Part used	References
Marapuama	*Ptychopetalum olacoides*	Leaf	(Oliveira et al. 2019)
Açaí	*Euterpe oleracea*	Fruit	(Barbosa et al. 2019)
Coramina	*Pedilanthus tithymaloides*	Leaf	(Vieira et al. 2022)
Embiratanha	*Pseudobombax marginatum*	Stem	(Menezes et al. 2015)
Camu‐camu	*Myrciaria dúbia*	Fruit	(Neves et al. 2015)
Embiratanha	*Pseudobombax marginatum*	Branches	(Menezes et al. 2015)
Amazon grape	*Pourouma cecropiifolia martius*	Fruit	(Lopes‐Lutz et al. 2010)
Embiratanha	*Pseudobombax marginatum*	Flower	(Menezes et al. 2015)
Bacaba	*Oenocarpus bacaba*	Fruit	(Dos Santos et al. 2015).
Amazon blueberries	*Clidemia hirta*	Fruit	(Assunção‐Júnior 2022)
Cocona	*Solanum sessiliflorum* Dunal	Fruit	(Lopes‐Lutz et al. 2010)
Cocoa	*Theobroma cacao* L.	Leaf	(de Araújo 2017)

It is important to emphasize that anthocyanins comprise a diverse group that includes six main structural types: pelargonidin, cyanidin, delphinidin, peonidin, petunidin, and malvidin (Rodriguez‐Amaya 2019). These compounds are found in purple, red, or black‐colored fruits, as well as in dark‐toned vegetables, as evidenced by Rodrigues et al. (2024). Among the most notable examples in the Amazonian context, the expressive presence of these molecules stands out in fruits such as açaí ( *Euterpe oleracea* ), bacaba (*Oenocarpus bacaba*), and camu‐camu (*Myrciaria dubia*), which, in addition to their coloring function, exhibit high antioxidant potential (de Jesus et al. 2020; Neves et al. 2015).

In summary, the studies analyzed not only confirm the presence of anthocyanins in plants, fruits, and flowers from the Amazon region but also highlight the growing recognition of the importance of these colorants in functional and bioactive contexts. Owing to its unparalleled biological diversity, the Amazon presents a promising scenario for the prospection of native fruits, many of which remain underexplored regarding their phytochemical composition and the antioxidant potential of their constituents (Cuéllar Álvarez et al. 2025).

An example that illustrates this scenario is the study conducted by Dos Santos et al. (2015), who investigated palm tree species whose fruits revealed significant sources of polyphenols and anthocyanins, in some cases with concentrations higher than those observed in conventionally consumed fruits. According to the authors, these compounds are strongly correlated with antioxidant activity, which reinforces the functional potential of these fruits, both for food applications and for the development of pharmaceutical products.

### 3.2 Carotenoids: Characteristics, Applications, and Natural Sources

Carotenoids are a class of natural colorants widely distributed in the plant kingdom, responsible for vibrant colors ranging from yellow, orange, and red. They are commonly found in foods such as carrots, pumpkins, mangos, tomatoes, peppers, papayas, and spinach, and belong to the group of terpenoid compounds recognized for their importance in both plant metabolism and human health (Zhao et al. 2022). In this scenario, it is important to highlight that their presence is associated not only with aesthetic functions but also with essential physiological processes in plants and humans (Viana et al. 2022).

Among the main representatives of this class, *α‐*carotene and *ß‐carotene* stand out. Both are fat‐soluble colorants synthesized in chloroplasts, where they play fundamental roles in the photosynthetic system, such as protection against oxidative stress caused by excess solar radiation and direct participation in the capture of light energy (Sun et al. 2020, 2022). According to the authors, unlike other colorants, carotenoids are directly involved in photochemical reactions, providing greater stability and efficiency to the processes of converting light into energy.

In addition to their occurrence in higher plants, these colorants are also produced by some fungi and bacteria. As discussed by Rapoport et al. (2021), this microbial synthesis can be understood as a defense mechanism against environmental variations, such as exposure to ultraviolet radiation or nutrient scarcity, a characteristic that expands the biotechnological possibilities of these compounds.

From a physiological perspective, carotenoids also act as precursors to biologically active substances. A prime example is abscisic acid (ABA), a plant hormone associated with the regulation of processes such as stomatal closure and seed dormancy induction (Auldridge and McCarty 2006). In the human body, *ß*‐carotene stands out, widely recognized as provitamin A. Its metabolic conversion is essential for eye health, especially in low light conditions, in addition to contributing to the proper functioning of the immune system, skin, and the prevention of diseases such as cataracts and age‐related macular degeneration (Eggersdorfer 2018).

Added to this is the fact that carotenoids exert a potent antioxidant action (Miranda et al. 2021). Han (2017) point out that these compounds are capable of neutralizing reactive oxygen species, helping to reduce the risk of chronic noncommunicable diseases such as cancer, cardiovascular disorders, and neurodegenerative diseases. In this context, regular consumption of fruits and vegetables rich in carotenoids has been associated with the prevention of nutritional deficiencies, especially hypovitaminosis A, a condition that still affects vulnerable populations in several regions of the world (Bas 2024).

Therefore, carotenoids are not restricted to the role of simple decorative colorants. As observed by Martins Cunha Junior et al. (2021), they are consolidated as multifunctional compounds, bringing together properties of nutritional, therapeutic, and industrial interest. From this perspective, the relevance of the Amazon as a potential producer of these bioactives stands out, especially considering the diversity of regional fruits with significant levels of carotenoids. Although there is still a gap in the investigation of these compounds in leaf structures, the data summarized in Table 2 demonstrate that different Amazonian fruit species contain significant concentrations of these colorants, reinforcing the need for further research and the valorization of endemic resources (Miranda et al. 2021).

**TABLE 2:** Amazonian plants identified as natural sources of carotenoids.

Common name	Scientific name	Part	References
Coramina	*Pedilanthus tithymaloides*	Leaf	(Vieira et al. 2022)
Peach palm	*Bactris gasipaes*	Fruit	(Chisté et al. 2021)
Pressed palm fiber	*Elaeis guineensis*	Fiber	(Prado et al. 2010)
Tucumã	*Astrocaryum vulgare*	Fruit	(Amorim et al. 2022)
Mussambê	*Tarenaya spinosa*	Leaf	(Vieira et al. 2022)
Buriti	*Mauritia flexuosa*	Fruit	(Santos et al. 2015)
Yellow mombin	*Spondias mombin*	Fruit	(Costa and Mercadante 2018)
Araçá‐boi	*Eugenia stipitata*	Fruit	(Garzón et al. 2012)
Inajá	*Maximiliana maripa* (Aubl.)	Fruit	(Anunciação et al. 2019)

**TABLE 3:** Amazonian plants identified as natural sources of betalains.

Common name	Scientific name	Part	References
Facheiro (ripe)	( *Pilosocereus pachycladus* RITTER)	Fruit	(Rodrigues et al. 2019)
Facheiro (unripe)	( *Pilosocereus pachycladus* RITTER)	Fruit	(Rodrigues et al. 2019)
*Tacinga inamoena*	(K. Schum.) [NP Taylor & Stuppy]	Fruit	(Dantas et al. 2016)
Dragon fruit	( *Hylocereus undatus* )	Peel	(Mello et al. 2015)
Dragon fruit	( *Hylocereus undatus* )	Peel	(Rodriguez et al. 2016)
*Tacinga inamoena*	(K. Schum.) [NP Taylor & Stuppy]	Peel	(Dantas et al. 2016)

### 3.3 Betalains: Characteristics, Applications, and Natural Sources

Betalains are characteristic colorants of species belonging to the order *Caryophyllales*, found in plant structures such as roots, flowers, and fruits (Leal‐Alcázar and Bautista‐Palestina 2025). They are nitrogenous compounds derived from the metabolism of tyrosine and betalamic acid, subdivided into two main groups: betaxanthins, which have colors ranging from yellow to orange, and betacyanins, responsible for hues ranging from red to violet (Polturak and Aharoni 2018).

As detailed by Deepak and Ahipa (2020) betaxanthins such as indicaxanthin and vulgaxanthin exhibit absorption peaks around 480 nm, while betacyanins such as betanin, isobetanin, neobetanin, and prebetanin absorb light around 540 nm. This spectral characteristic reinforces their potential use as high‐intensity natural dyes. In addition to providing attractive coloration, betalains have several bioactive properties. According to Gengatharan and Dykes (2016), these compounds demonstrate significant antioxidant activity, in addition to potential antimicrobial, anti‐inflammatory, antitumor, lipid‐lowering, antidiabetic, and chemopreventive effects.

According to Sadowska‐Bartosz and Bartosz (2021), the presence of carboxyl groups in the molecular structure of betalains may be related to their chemical stability and biological efficacy, contributing to functional applications in food and pharmaceuticals. However, the occurrence of these colorants in Amazonian species remains underexplored. Although there are gaps in the literature regarding the presence of betacyanins in plants native to the Brazilian Amazon, promising evidence has already been observed in several fruits from the region, as shown in Table 3 of this study.

According to Carvalho et al. (2017), this trend indicates significant potential for harnessing Amazonian species as natural sources of betacyanins. In addition to their antioxidant properties, the vibrant hues of these fruits are attracting interest from the food and beverage industry, particularly as natural and progressive alternatives to synthetic dyes. In the international market, attributes such as flavor, appearance, and nutritional value confer a competitive advantage to these bioactive ingredients sourced from Amazonian biodiversity.

The stability of betalains, in turn, is strongly associated with the pH of the medium and environmental conditions. In environments with a pH between 4 and 5, these colorants appear reddish‐violet. However, at more alkaline pH, they acquire bluish hues and become more susceptible to degradation. According to Mello et al. (2015), the stability of betalains is best preserved between pH 3.2 and 7, with marked degradation observed at extreme pHs, such as 2.4 and 8. These colorants are water‐soluble but sensitive to light, heat, oxygen, and the presence of metal ions, which limits their use in formulations requiring long‐term stability. Even so, its performance is superior to that of anthocyanins in slightly acidic or neutral pH ranges (Mello et al. 2015).

Corroborating these observations, Gengatharan and Dykes (2016) tested the stability of betacyanin extracted from red dragon fruit ( *Hylocereus polyrhizus* ) in dairy formulations. The results were compared to conventional dye extracted from beetroot ( *Beta vulgaris* ) and demonstrated that dragon fruit betacyanin has greater thermal resistance and stability during refrigerated storage. Specifically, samples treated at 50°C and 85°C showed less degradation, demonstrating their robustness to heat.

During the 10‐week storage period at 4°C, both sources showed color changes. However, the dragon fruit betacyanin remained more stable in terms of colorant content and color preservation in milk, demonstrating superior performance. The authors also observed a moderate antimicrobial effect in samples treated with dragon fruit color, indicating a potential additional benefit for preservation. From a sensory perspective, the color provided by dragon fruit was better accepted by consumers (average score of 3.89 ± 1.89) compared to E‐162 (6.10 ± 1.71), suggesting that the mildness of the color positively influences the perception of the product's naturalness.

These findings are in line with the study by Vaillant et al. (2020), which highlighted the technological and functional use of dragon fruit betacyanins as a promising alternative to the use of synthetic dyes, especially in formulations subjected to heat treatment, such as dairy products and ready‐to‐drink juices.

At the same time, research focused on the bromatological characterization of Amazonian fruits has reinforced this scenario. Studies such as those by Rodrigues et al. (2019), who analyzed the facheiro (*Pilosocereus* spp.), Rodriguez et al. (2016), who investigated the white dragon fruit ( *Hylocereus undatus* ), and Dantas et al. (2016), who studied the quipa ( *Tacinga inamoena* ), showed significant levels of total betalains in both the pulp and peel of these species, demonstrating their potential for the development of new functional ingredients.

It is believed that the rational use of Amazonian biodiversity, combined with sustainable extraction and processing practices, can significantly contribute to the advancement of the natural dye bioindustry. By promoting the use of native species, respecting traditional knowledge and ecological limits, it becomes possible to develop innovative, healthier products with greater ecological appeal, aligned with consumer demands (Table 3).

### 3.4 Chlorophylls: Characteristics, Applications, and Natural Sources

Chlorophylls, present in leaves, fruits, algae, and some bacteria, play an essential role in photosynthetic organisms. These green colorants are responsible for capturing sunlight and transforming it into chemical energy through photosynthesis. According to Kräutler (2014), their absorption occurs primarily in the blue and red bands of the visible spectrum, while green light is reflected, which is why most plants exhibit a greenish coloration. During fruit ripening or leaf senescence, chlorophylls are progressively degraded, a process that results in the formation of specific catabolites and alters the appearance of plant tissue (Gorfer et al. 2022).

Structurally, chlorophylls are divided into two major groups based on their physicochemical properties. The first is composed of chlorophylls *a* and *b*, which, due to their esterified phytol chain, are nonpolar and predominate in the chloroplasts of higher plants. The second group includes chlorophylls C_1_, C_2_, and C_3_, characterized by the absence of phytol and, consequently, greater polarity and affinity with marine organisms such as algae and phytoplankton. This differentiation directly influences solubility, subcellular localization, and light energy absorption performance (Lefebvre et al. 2020).

Research conducted in the Amazon has explored variations in chlorophyll levels in native species, both in plants and fruits (Table 4). Studies such as those by Andrade Júnior et al. (2016) and Leonardo et al. (2013) seek to understand how environmental variables such as fertilization, nutrient availability, and light intensity influence the production and stability of these biomolecules. As argued by Lamarre et al. (2012), seasonal variations in leaf chlorophyll (SPAD/estimates) in Amazonian species under different light conditions are essential to understanding such dynamics and thus assessing the impact of ecological factors, including climate change and anthropogenic disturbances, on pigment biosynthesis.

**TABLE 4:** Amazonian plants identified as natural sources of chlorophylls.

Common name	Scientific name	Part	References
Araça‐boi	*Eugenia stipitata*	Fruit	(Barros et al. 2017)
Inga	*Inga laurina* (SW) Wild.	Leaf	(Lamarre et al. 2012)
Swartzia	*Swartzia apetala* Raddi.	Leaf	(Lamarre et al. 2012)
	*Bombacoideae*	Leaf	(Lamarre et al. 2012)
Achachairu	*Reedia laterifolia* L.	Fruit	(Barros et al. 2017)
White biriba	*Eschweilera*	Leaf	(Mori et al. 2019)
Oiticica	*Licania*	Leaf	(Mori et al. 2019)
Munguba	*Pseudobombax munguba*	Leaf	(Marenco et al. 2019)
Cubiu	*Solanum sessiliflorum*	Fruit	Andrade Júnior et al. (2016)
Cubiu	*Solanum sessiliflorum*	Fruit	(Andrade Júnior et al. 2016)
Bacaba	*Oenocapus bacaba*	Fruit	(Barros et al. 2017)

In this regard, several authors emphasize that plant adaptive responses can be decisive for the regulation of photosynthesis and, consequently, for the accumulation of bioactive compounds. This also opens up possibilities for the application of extracted colorants, especially in contexts that value Amazonian biodiversity as a biochemical and ecological asset (Mori et al. 2019; Marenco and de Freitas Sousa 2019; Lamarre et al. 2012; Ávila‐Lovera et al. 2017; Ávila‐Lovera 2024).

Mori et al. (2019), in turn, demonstrated that chloroplast content may not vary significantly depending on water availability. To this end, the authors evaluated 12 Amazonian species in two areas, including a nutrient‐rich whitewater floodplain forest and a nutrient‐poor blackwater floodplain forest (igapó). According to the authors, 11 characteristics important for the carbon and nutrient balance of trees were measured based on comparative analysis between leaves from different exposures, which showed no changes in leaf chlorophyll content.

These results converge with the findings of Andrade Júnior et al. (2016), who observed significant variations in the levels of chlorophyll *a* and *b*, higher in the green peel, and a decline during the ripening of the Amazonian fruit *Cubiu*, after analysis of these compounds by HPLC for pigmentation indices. In this context, it becomes evident that the conditions of the fruit's ripening stage can influence chlorophyll biosynthesis and represent relevant results for other researchers working with pigment extraction.

Therefore, when considering the collection of raw materials for biotechnological, industrial, or academic purposes, it is crucial to understand how ecological and physiological factors modulate the presence and concentration of chlorophylls. These variables not only affect the quality and stability of extracts but also contribute to the development of more sustainable and efficient protocols, respecting the specific characteristics of Amazonian species and their environmental context.

## 4 Extraction Methods

### 4.1 Green Solvents and Modern Extractions

In the current panorama of food science, the search for environmentally responsible alternatives has driven the adoption of *green solvents*, also called *ecological solvents*. These solvents are distinguished by their low toxicity, high biodegradability, and their renewable origin. Thus, they offer not only a lower environmental impact but also greater safety in applications related to the food production chain and public health (Ling 2022). This approach is in line with the growing call for more sustainable and cleaner technologies, demonstrating a paradigm shift in the way inputs are selected and used in the extraction stages of bioactive compounds.

In the field of pigment extraction, there has been a progressive increase in the adoption of green methodologies as a technological route for obtaining natural compounds from plants (Osamede Airouyuwa et al. 2024; Tzanova et al. 2024). (Table 5) Recent studies, such as that by Morón‐Ortiz et al. (2024), demonstrate that the use of natural eutectic mixtures, such as menthol/camphor (1:1), combined with the ultrasound technique, provided not only high yields but also greater stability in the process of extracting carotenoids from orange peel ( *Citrus sinensis* ). In the same study, the authors investigated the extraction of lutein from *Tagetes erecta* flowers using the bio‐based solvent 2‐methyltetrahydrofuran (2‐MeTHF), achieving a 12% higher performance than hexane, in addition to a lower environmental impact, thus reinforcing the applicability of these alternative systems.

**TABLE 5:** Studies with green solvents in the extraction of natural colorants associated with modern techniques.

Matrix	Target colorants	Green solvent	Technique	Extraction scale	Highlight
Pumpkin	*ß*‐carotene	NADES (caprylic/capric acid)	Ultrasound	Laboratory	(Ling et al. 2022)
Orange peel	Various carotenoids	NADES menthol/camphor	Ultrasound	Laboratory	(Morón‐Ortiz et al. 2024)
Tagetes flowers	Lutein	2‐MeTHF	Maceration	Laboratory	(Morón‐Ortiz et al. 2024)
Apricot	*ß* ‐carotene	DES (choline chloride/tartaric acid)	Grinding + ethanol co‐solvent	Laboratory	(Viñas‐Ospino et al. 2023)
Jabuticaba	Anthocyanins	ChCl: Pro and ChCl: Ma	Pressurized liquid extraction (PLE)	Laboratory	(Benvenutti and Zielinski 2022)
Beetroot	Betacyanins and betaxanthins	Various DES (ChCl: glucose, etc.)	Optimized by RSM	Laboratory	(Kaba et al. 2024)
*Spirulina platensis*	Carotenoids + chlorophylls	NADES (glucose/glycerol/water and menthol/lactic acid)	Ultrasound with RSM	Laboratory	(Martins et al. 2023)
Carrot House	Carotenoids	Supercritical CO_2_ + EtOH	SFE‐CO_2_ (dynamic extraction) + EtOH (co‐solvent)	Laboratory	(Andrade Lima et al. 2018)
Vegetable residues	Carotenoids	Supercritical CO_2_ + EtOH	SFE‐CO_2_ (dynamic extraction) + EtOH (co‐solvent)	Laboratory	(Andrade Lima et al. 2019)
Tomato	Lycopene	Supercritical CO_2_	Soxhlet extraction	Laboratory	(Popescu et al. 2022)
Yeast (*Rhodotorula* spp.)	Carotenoids	Supercritical CO_2_ + co‐solvent EtOH	Applied Separations Spe‐ed SFE‐2 (bench top)	Laboratory	(Larocca et al. 2025)

Different studies have established systematic comparisons between conventional solvents and ecological alternatives in order to demonstrate the feasibility and efficiency of these new approaches. For instance, Viñas‐Ospino et al. (2023) employed a Deep Eutectic Solvent (DES), deep eutectic solvents generally obtained from the combination of natural compounds such as quaternary ammonium salts and organic acids, known for being biodegradable, low‐cost, and low‐toxicity, composed of choline chloride and tartaric acid to extract carotenoids from apricot. The method yielded 41.3 mg/100 g of plant material, a performance significantly higher than that obtained with ethanol as a co‐solvent (11.5 mg/100 g). These findings highlight that sustainable practices not only reduce environmental impacts but can also enhance the efficiency of extraction processes.

In the same perspective, proposed an approach based on pressurized liquid extraction (PLE) using DES formulated with choline chloride (ChCl) and propylene glycol or maleic acid, focusing on obtaining anthocyanins from jabuticaba. The EcoScale certification values were 72.54 for ChCl: Pro and 69.45 for ChCl: Ma, and were higher than those obtained with water or ethanol, demonstrating the technical and environmental superiority of the formulation. Similarly, Kaba et al. (2024) applied several choline chloride‐based DES to extract betalains from beetroot, highlighting the ChCl: glucose formulation (DES 5), which provided extraction of ~1192 mg/kg of total colorants, with high selectivity under mild conditions (30°C, molar ratio 1:0.75).

On the other hand, for researchers such as Chevé‐Kools et al. (2025), Natural Deep Eutectic Solvents (NADES) represent an even more promising trend, as they are composed exclusively of natural metabolites such as sugars (glucose, fructose), organic acids (citric, lactic, malic), amino acids (proline, glycine), alcohols and polyols (glycerol, sorbitol), and nitrogenous bases (urea, choline), all classified as safe for consumption (GRAS). According to Chevé‐Kools and collaborators, NADES have amphiphilic properties, enabling the efficient solubilization of polar and nonpolar compounds simultaneously, which makes them highly versatile in the extraction of mixed colorants.

In this regard, Martins et al. (2023) demonstrated the efficiency of NADES in the integrated extraction of colorants such as carotenoids and chlorophylls from the microalga *Spirulina platensis*. Using glucose/glycerol/water‐based systems, the authors demonstrated improved performance in ultrasound‐assisted extractions. This formulation demonstrated the highest total dye productivity compared to the traditional aqueous solvent. This performance is explained by the high degree of compatibility of NADES with biomass constituents and the synergy promoted by ultrasonic cavitation, which facilitates colorants' release.

Based on the evidence presented, it can be inferred that the use of green solvents, particularly DES and NADES, has emerged as a viable, innovative, and environmentally responsible alternative for the extraction of natural colorants. Although most of the studies cited have focused on plant species outside the Amazon, these sustainable methodologies show great potential for the extraction of colorants from Amazonian species, whose chemical diversity remains largely unexplored. The wide variety of native plants in the region provides a rich source of bioactive compounds, making the use of green solvents in combination with modern extraction techniques a gateway to new experiments and the development of innovative bioproducts. Thus, the implementation of these approaches enables better utilization of local resources and contributes to the valorization of Amazonian biodiversity in an environmentally conscious and technologically advanced manner.

The studies conducted by Andrade Lima et al. (2018, 2019)` present a sequential and complementary approach to the application of supercritical CO_2_ extraction (SFE) for the recovery of carotenoids from vegetable residues. In the 2018 study, the authors focused on optimizing and modeling the process using carrot peel as a model matrix, systematically evaluating temperature, pressure, and ethanol concentration as a co‐solvent, demonstrating rapid extraction kinetics (97% in 30 min), high recovery, and the scalability potential of the process. The 2019 study, in turn, expanded the application of the previously validated model, using the optimized conditions on 15 different carotenoid‐rich vegetable matrices, confirming the robustness and reproducibility of the method, with recoveries exceeding 90% for most samples and particularly efficient extraction of ß‐carotene. Together, the two studies demonstrate a consistent methodological evolution: first establishing and validating an optimized model at laboratory scale and subsequently demonstrating its broad applicability to different agro‐industrial residues, reinforcing the potential of SFE as a green and versatile technology for valorizing dye‐rich by‐products.

The analysis developed by Popescu et al. (2022) corroborates the results of Larocca et al. (2025) as it allows for a relevant comparative analysis regarding the efficiency of supercritical CO_2_ extraction (SFE‐CO_2_) in matrices of different natures. Upon analyzing the results, it can be inferred that the physicochemical composition of the matrix is a determining factor for process performance. In the research by Popescu et al. (2022) on tomato slices, the incorporation of oilseeds as modifiers promoted a significant increase in mass transfer and carotenoid solubilization, raising the extract yield from 66.00 to 108.65 g/kg of dry sample and allowing the obtainment of fractions highly concentrated in lycopene. From these data, it is observed that plant matrices with higher lipid content favor the solubility of carotenoids in the supercritical fluid, especially under conditions of high pressure (450 bar) and temperature (70°C).

On the other hand, when examining the results obtained by Larocca et al. (2025), it is verified that in Rhodotorula spp. microbial biomass, the process efficiency proved to be more dependent on the addition of co‐solvent than on increasing temperature, achieving maximum recoveries close to 74%. This implies that, in complex cellular matrices, the structural barrier of the cell wall limits solvent diffusion, requiring intensification strategies such as pre‐treatment and modification of the extraction system's polarity. Thus, the comparative analysis between the groups indicates that while lipid‐rich vegetable residues show higher overall extract yield and high concentration of dyes, microbial matrices require operational adjustments to maximize recovery.

Consequently, it is generally observed that SFE‐CO_2_ reveals high versatility and a sustainable character; however, its efficiency is directly related to the structural and compositional characteristics of the processed matrix. Thus, the results of Larocca et al. (2025) show that strategies such as lipid modification of the plant matrix or the use of co‐solvent in microbial biomass are decisive for optimizing carotenoid recovery.

### 4.2 Conventional Solvent Extraction

The use of organic solvents such as methanol, ethanol, and acetone has been widely employed for the extraction of colorant compounds, given their ability to solubilize nonpolar substances that are generally not efficiently extracted by water (Song et al. 2018). According to the specialized literature, this traditional approach remains relevant, although it is not without its limitations. Among the challenges is limited yield, which has driven the development of auxiliary techniques that enhance the effectiveness of these solvents. Faced with this problem, techniques such as maceration are being explored.

As discussed by Wojdyło et al. (2021), this technique weakens cell walls, disrupting cytoplasmic membranes and facilitating the passage of solvent into the plant matrix. This condition results in more efficient extraction and a higher yield of compounds of interest.

Regarding widely used solvents, water emerges as a promising alternative, especially in the extraction of polar and water‐soluble compounds (Liu et al. 2019). However, as pointed out by Saini and Keum (2018), its effectiveness is limited when compared to nonpolar molecules, which justifies its combined use with other organic solvents, such as ethanol. This combination favors the rupture of the cell wall and expands the spectrum of extracted compounds.

Several studies have investigated strategies to optimize the extraction of specific colorants (Table 6). Yao et al. (2015), for example, evaluated the application of a macroporous polymeric adsorbent in the purification of anthocyanins from *Vaccinium myrtillus* L., achieving a purity of 96%. Similarly, Heras and Alvis (2013) explored the extraction of anthocyanins from eggplant ( *Solanum melongena* L.) using acidified ethanol at concentrations between 50% and 90%, varying the time (4–12 h) and temperature (30°C–60°C). The best results were obtained with 50% acidified ethanol at 30°C for 4 h, achieving 62 mg/100 g of anthocyanins.

**TABLE 6:** Characteristics of solvent systems used in the extraction of natural colorants: Food suitability, risks, and limitations.

^1^Solvent system	^1^Suitability for type of colorant	^1^Main limitations	^2^Suitability for contact with food	^2^Main risks	References
Water	Betalains; Anthocyanins (auxiliary)	Low yield for nonpolar colorants; may promote enzymatic hydrolysis	GRAS	No relevant toxicological risk	^1^Zin and Bánvölgyi (2023); ^2^ANVISA/FDA
Acidified water (Water–HCl)	Anthocyanins; Betalains	Requires neutralization/acid removal step; risk of degradation due to extreme pH	Approved with restrictions; residual HCl must be removed	Corrosivity at high HCl concentrations	^1^Oliveira et al. (2020); ^2^ANVISA/FDA
Water/ethanol 1:1 (v/v)	Betalains; Anthocyanins	Variable yield depending on proportion; need for removal of residual ethanol	Approved, provided it is food grade	Moderate flammability	^1^Gómez‐López et al. (2022); ^2^ANVISA/FDA
Ethanol 95%	Phenolic compounds; Anthocyanins; Carotenoids	Reduced efficiency for highly nonpolar colorants without nonpolar co‐solvent	Approved, provided it is food grade	Flammability; low systemic toxicity	^1^Khoo et al. (2017); ^2^ANVISA/FDA
Ethanol 95% + HCl (1%)	Anthocyanins	Higher stability, but additional purification step required	Approved, with restrictions; residual HCl must be controlled	Corrosivity of HCl; flammability of ethanol	^1^Khoo et al. (2017); ^2^ANVISA/FDA
Ethanol 50%	Anthocyanins; Phenolic compounds	Lower efficiency for nonpolar compounds; may co‐extract polar interferents	Approved, provided it is food grade	Low to moderate flammability	^1^Leichtweis et al. (2019); ^2^ANVISA/FDA
Ethanol 20%	Anthocyanins (high solubility in diluted medium)	Lower selectivity; risk of microbiological degradation of the extract	Approved, provided it is food grade	Very low flammability	^1^Yao et al. (2015); ^2^ANVISA/FDA
Methanol 95%	Phenolic compounds; Anthocyanins; Chlorophylls	Restricted to laboratory analyses; requires specialized disposal; not scalable for foods	Prohibited in foods, analytical use only	High systemic toxicity (blindness, death at high doses); high flammability	^1^Khoo et al. (2017); ^2^ANVISA/FDA
Methanol 95% + HCl (1%)	Anthocyanins (potent and selective extraction)	Restricted to qualitative/quantitative analysis; not applicable to the food industry	Prohibited in foods, analytical use only	High toxicity; corrosivity; high flammability	^1^Khoo et al. (2017); ^2^ANVISA/FDA
Methanol 50%	Phenolic compounds; Anthocyanins	Same as pure methanol; co‐extraction of water‐soluble interferents	Prohibited in foods, analytical use only	Moderate to high systemic toxicity; flammability	^1^Khoo et al. (2017); ^2^ANVISA/FDA
Acetone (100%)	Chlorophylls (a and b); Nonpolar carotenoids	May convert chlorophylls into chlorophyllides; residual traces compromise food safety	Prohibited in foods, analytical use only	High flammability; mucosal irritation; mild neurotoxicity	^1^Hu et al. (2013); ^2^ANVISA/FDA
Hexane	Carotenoids (ß‐carotene, lycopene); Pigmented lipids	Petrochemical solvent; not green; toxic residue requires rigorous recovery	Prohibited in foods, analytical use only	High flammability; neurotoxicity (prolonged use)	^1^Prado et al. (2010); ^1^Amorim et al. (2022); ^2^ANVISA/FDA
DES/NADES (e.g., ChCl:glucose; menthol/camphor)	Anthocyanins; Betalains; Carotenoids; Chlorophylls	High viscosity makes filtration difficult; synthesis process must be controlled; few long‐term safety data	Approved; NADES are GRAS; DES depends on components	Variable according to composition; generally low toxicity	^1^Kaba et al. (2024); ^1^Morón‐Ortiz et al. (2024); ^1^Benvenutti et al. (2022); ^2^ANVISA/FDA
Supercritical CO_2_ (SFE) ± ethanol co‐solvent	Carotenoids (total); Pigmented lipids	High equipment cost; limited to nonpolar compounds; complex scale‐up	Approved; CO_2_ leaves no residues; ethanol as co‐solvent is approved	Risk of high pressure (industrial operation); CO_2_ asphyxiant in confined spaces	^1^Oliveira et al. (2020); ^1^Prado et al. (2010); ^2^ANVISA/FDA

*Note:* ANVISA, Brazilian Health Regulatory Agency (Agência Nacional de Vigilância Sanitária); ChCl, cloreto de colina; DES, Deep Eutectic Solvent; GRAS, Generally Recognized As Safe (FDA); NADES, Natural Deep Eutectic Solvent; SFE, Supercritical Fluid Extraction.

Compared to conventional methods, Leichtweis et al. (2019) highlighted the superiority of ultrasound‐assisted extraction when evaluating colorants from *Prunus spinosa* L. The researchers tested varying dilutions of ethanol and different operating conditions. The ultrasonic method with 5 min of extraction, 400 W of power, and 47.98% ethanol resulted in an anthocyanin content of 18.17 mg/g in the base extract.

The discussion on hydroethanolic solvents is also addressed by Gómez‐López et al. (2022), who, using a 1:1 (v/v) ratio and a temperature of 25°C, managed to extract betalains from *Opuntia stricta var. dillenii* . Pressurized liquid extraction (PLE), in this case, presented yields similar to those of conventional methods, with emphasis on bioactives such as neobetanin and piscidic acid.

Regarding chlorophyll stability, Hu et al. (2013) point to the conversion of these molecules into chlorophyllides as a recurring challenge, especially when using solvents such as 80% acetone in chromatographic procedures (HPLC). Suppression of phytol during extraction compromises accurate quantification of colorants. To mitigate this conversion, the authors tested methods such as boiling the leaves for 5 min, grinding at −20°C, and extraction with N,N′‐dimethylformamide (DMF), achieving good results in *Arabidopsis thaliana* .

Additionally, Oliveira, Minuzzi, et al. (2020a); Oliveira, Schottroff, et al. (2020b) tested the application of pulsed electric fields (PEF) associated with the use of conventional solvents in the extraction of carotenoids from dried yeasts of *Rhodotorula glutinis* . The authors observed that ethanol presented the best performance, being able to extract more than 80% of the carotenoids after PEF treatment followed by incubation in aqueous buffer for 24 h. In addition to high efficiency, the process stands out for being clean, selective, and environmentally sustainable.

Along the same lines, the study by Zin and Bánvölgyi (2023) stands out for prioritizing the exclusive use of water as a solvent in the extraction of colorants from beetroot peel ( *Beta vulgaris* L.) through microwave‐assisted extraction technology (MAE). Even without the addition of co‐solvents, the authors obtained significant amounts of betalains, betacyanins, and betaxanthins after 150 s of MAE at 800 W, which reinforces the potential of green methods.

Although highly efficient in extracting chlorophylls, pure methanol is highly toxic, which restricts its use in food, but keeps it as an option in industrial applications, such as textiles. Amr et al. (2022) analyzed the extraction of anthocyanins from black grape pomace using different solvent purities. Their findings revealed that pure methanol was significantly more efficient than ethanol, resulting in higher levels of extracted anthocyanins.

In contrast, pure ethanol has emerged as a viable and safe alternative for extracting colorants, especially carotenoids, when combined with modern techniques such as ultrasonication (Ribeiro et al. 2010). In the study by Ribeiro et al. (2010), the use of ethanol as the sole solvent resulted in effective extraction of carotenoids from the pulp of *Mauritia flexuosa* L., with high yield and well‐defined fractions.

For anthocyanins, although absolute ethanol is not the most commonly used, solutions acidified with HCl (1%) or hydroethanolic mixtures (50%–70%) have shown more expressive results (Khoo et al. 2017; Heras and Alvis 2013). In this context, Araújo et al. (2023) demonstrated the effectiveness of 85% acidified ethanol (85:15 EtOH: HCl) for the extraction of anthocyanins from red cabbage, preserving the qualitative profile of the colorants. These findings are in agreement with the studies of Silva et al. (2023), who compared different methodologies and confirmed the equivalence of extraction with ethanol compared to other solvents.

In addition to punctual performance in certain systems, review studies have shown that green solvents have significant advantages over conventional ones (Osamede Airouyuwa et al. 2024; Tzanova et al. 2024). While solvents like ethanol, methanol, acetone, and hexane are widely used, they offer risks of toxicity, flammability, and high energy demand for recovery. Green solvents, such as Deep Eutectic Solvents (DES), natural ionic liquids, and supercritical fluids, stand out for being biodegradable, less toxic, low cost, and, in many cases, for providing greater selectivity and yield (Viñas‐Ospino et al. 2023). For example, Morón‐Ortiz et al. (2024) reported that carotenoid extraction using DES achieved yields up to 3.5 times higher than ethanol, in addition to requiring less solvent volume and shorter processing time. This type of comparison reinforces that the adoption of green methodologies is not limited to environmental appeal but also offers quantitative and technological gains compared to traditional approaches.

## 5 Conclusion

Although there is a wide variety of records for anthocyanins and carotenoids, research on betalains and chlorophylls is still in its infancy, revealing important gaps to be explored. Furthermore, it was noted that most studies focus on fruits, while vegetative structures such as leaves, stems, and seeds remain underutilized, representing a promising field for future research. Another point observed is that, despite advances in the use of sustainable methodologies, there are still few studies that apply green solvents in comparison to conventional ones in Amazonian matrices, which limits the consolidation of standardized protocols for the region. In this context, new research can advance both the phytochemical characterization of understudied species and the optimization of extraction techniques, prioritizing sustainable routes that preserve the colorants' bioactivity. Integrating these perspectives can strengthen the regional bioeconomy and add value to local production chains, promoting safe alternatives to synthetic dyes and meeting the growing demand for natural ingredients. Thus, this type of study contributes not only to the scientific appreciation of biodiversity but also to the creation of economic and technological opportunities in the Amazon, consolidating it as a strategic patrimony for the development of bioproducts with a positive impact on the food, pharmaceutical, and cosmetics industries.

## Author Contributions

Zandleme Oliveira: conceptualization, hypothesis development, data collection, writing – original draft. Antônio Quaresma: investigation, data curation, writing, review, and editing. Kamila Vasconcelos: investigation, data curation, writing, review, and editing. Amanda Mumberger: translation, writing, review, and editing. Leomara Silva: writing, review, and editing. Ricardo Oliveira: writing, review, and editing. Rosa Mourão: writing, review, and editing. Sandra Sarrazin: writing, review, and editing.

## Funding

This work was supported by Coordenacao de Aperfeicoamento de Pessoal de Nivel Superior.

## Ethics Statement

This study does not involve any human or animal testing.

## Consent

Written informed consent was obtained from all study participants.

## Conflicts of Interest

The authors declare no conflicts of interest.

## Data Availability

No new data were created or analyzed in this study. Data sharing is not applicable as this article is a review based on previously published studies.
